# O3-3 Active commuting and healthy behavior among adolescents in neighborhoods with varying socioeconomic status

**DOI:** 10.1093/eurpub/ckac094.019

**Published:** 2022-08-29

**Authors:** Benti Geleta Buli, Annika Tillander, Terence Fell, Katarina Bälter

**Affiliations:** 1 Division of Public Health Sciences, Mälardalen University, Västerås, Sweden; 2 Department of Computer and Information Science, Linköping University, Linköping, Sweden; 3 Division of Political Science, Mälardalen University, Västerås, Sweden; 4 Department of Medical Epidemiology and Biostatistics, Karolinska Institutet, Stockholm, Sweden

**Keywords:** active commuting, physical activity, socioeconomic status, lifestyle

## Abstract

The World Health Organization recommends active commuting to and from school as a source of physical activity. Worldwide, active commuting, i.e. walking or biking, is determined by various factors including gender, socioeconomic status of families and neighborhoods, the built envormnet, distance to schools, perceived neighborhood safety, life styles, and availability of walkways, biking paths and parks. Previous studies from Swedish indicate that the proportion of youth being active commuters descres with age, but has also decresed over time. This study aimed at assessing health and environmental factors associated with modes of transportation to and from school among 333 adolescents aged 16-19 living in a middle-sized city in Sweden.

The present city has 380 kilometer of walking and biking paths that cover the whole city, of which parts are heated in the winter to avoid snow and ice. Still, only 32% of the adolescents were active commuters to and from school. Active commuting was linked to other healthy behaviours including more frequenct daily consumption of fruits and vegetables and less frequent consumption of junk food. Morover, the presence of well illuminated walking and biking paths was strongly associated with active commuting, as compared to not having access to less illuminated paths. However, the relation between active commuting and socioeconomic status of the neighborhood was complex and needs to be explored in future larger studies.

The results will be used to increase awareness of the health and enviromental benfits of active commuting among adolencnets, schools, and local policy makers.

